# The inhibitor apoptosis protein antagonist Debio 1143 Is an attractive HIV-1 latency reversal candidate

**DOI:** 10.1371/journal.pone.0211746

**Published:** 2019-02-04

**Authors:** Michael Bobardt, Joseph Kuo, Udayan Chatterji, Sumit Chanda, Susan J. Little, Norbert Wiedemann, Gregoire Vuagniaux, Philippe A. Gallay

**Affiliations:** 1 Department of Immunology & Microbiology, The Scripps Research Institute, La Jolla, California, United States of America; 2 Infectious and Inflammatory Disease Center, Sanford Burnham Prebys Medical Discovery Institute, La Jolla, United States of America; 3 Department of Medicine, University of California, San Diego, California, United States of America; 4 Debiopharm International S.A., Lausanne, Switzerland; University of Pittsburgh, UNITED STATES

## Abstract

Antiretroviral therapy (ART) suppresses HIV replication, but does not cure the infection because replication-competent virus persists within latently infected CD4+ T cells throughout years of therapy. These reservoirs contain integrated HIV-1 genomes and can resupply active virus. Thus, the development of strategies to eliminate the reservoir of latently infected cells is a research priority of global significance. In this study, we tested efficacy of a new inhibitor of apoptosis protein antagonist (IAPa) called Debio 1143 at reversing HIV latency and investigated its mechanisms of action. Debio 1143 activates HIV transcription via NF-kB signaling by degrading the ubiquitin ligase baculoviral IAP repeat-containing 2 (BIRC2), a repressor of the non-canonical NF-kB pathway. Debio 1143-induced BIRC2 degradation results in the accumulation of NF-κB-inducing kinase (NIK) and proteolytic cleavage of p100 into p52, leading to nuclear translocation of p52 and RELB. Debio 1143 greatly enhances the binding of RELB to the HIV-1 LTR. These data indicate that Debio 1143 activates the non-canonical NF-kB signaling pathway by promoting the binding of RELB:p52 complexes to the HIV-1 LTR, resulting in the activation of the LTR-dependent HIV-1 transcription. Importantly, Debio 1143 reverses viral latency in HIV-1 latent T cell lines. Using knockdown (siRNA BIRC2), knockout (CRIPSR NIK) and proteasome machinery neutralization (MG132) approaches, we found that Debio 1143-mediated HIV latency reversal is BIRC2 degradation- and NIK stabilization-dependent. Debio 1143 also reverses HIV-1 latency in resting CD4+ T cells derived from ART-treated patients or HIV-1-infected humanized mice under ART. Interestingly, daily oral administration of Debio 1143 in cancer patients at well-tolerated doses elicited BIRC2 target engagement in PBMCs and induced a moderate increase in cytokines and chemokines mechanistically related to NF-kB signaling. In conclusion, we provide strong evidences that the IAPa Debio 1143, by initially activating the non-canonical NF-kB signaling and subsequently reactivating HIV-1 transcription, represents a new attractive viral latency reversal agent (LRA).

## Introduction

According to estimates by WHO and UNAIDS, approximately 40 million people are currently living with HIV-1. According to the latest estimates from the Centers for Disease Control and Prevention, 38,500 people became newly infected with HIV-1 in the United States in 2015, and 2.1 million worldwide [1]. Antiretroviral therapy (ART) represses HIV-1 replication and stops disease progression, allowing infected people to live with the infection [2]. Yet, ART does not eliminate the infection since replication-competent HIV-1 survives in latently infected CD4+ T cells during many years of ART [3–5]. Resting CD4+ T cells harbor integrated viral genomes and serve as permanent source of *de novo* infectious viruses. Long-term ART is accompanied with issues including health problems due to chronic drug exposure, expensive cost and stringent compliance requirement [6]. Thus, new strategies to eradicate these viral reservoirs represent an utmost clinical priority.

Several strategies for eradicating latent HIV-1 reservoirs have been envisioned [7]. A promising strategy is termed “kick and kill”. Since HIV-1 latent cells express low to no viral proteins, they cannot be directly killed by viral cytopathic effects or by immune response recognition such as cytotoxic T lymphocytes (CTL) or natural killer (NK) cells, which need viral protein expression to detect infected cells [8–10]. However, triggering of viral protein expression (*kick*) in latently HIV-1-infected cells should restore their vulnerability to virus-mediated cytopathic killing and/or immune defense-mediated killing (*kill*). It remains to be determined whether triggering expression of latent HIV-1 in patients (*kick*) will suffice to eliminate (*kill*) latent cellular reservoirs, or whether the immune defense will be needed [8–10]. Interestingly, the Zack lab recently demonstrated that a synthetic bryostatin-1 analog alone was able to induce both “kick” and “kill” responses in latently HIV-1-infected CD4+ T cells in humanized bone marrow/liver/thymus (BLT) mice [11].

Several latency reversal agents (LRAs) have been identified [2, 12–16] such as protein kinase C (PKC) modulators including prostratin, ingenol esters and bryostatin-1 [17–18].

However, tumorigenesis and other adverse side effects (i.e., cytokine overexpression) have been observed in patients treated with PKC modulators [19]. Histone deacetylase (HDAC) inhibitors such as panobinostat and vorinostat represent another promising class of LRAs [20]. Although HDAC inhibitors showed some degree of reactivation *in vitro* in HIV-1 latent cell lines, their efficacy in HIV-1 patients, infected BLT mice and *ex vivo*, is relatively poor [21–24]. Thus, identification of new safe and potent LRAs represents a critical unmet need in order to design novel pharmacological strategies to eradicate the viral latent reservoir.

**Fig 1:**
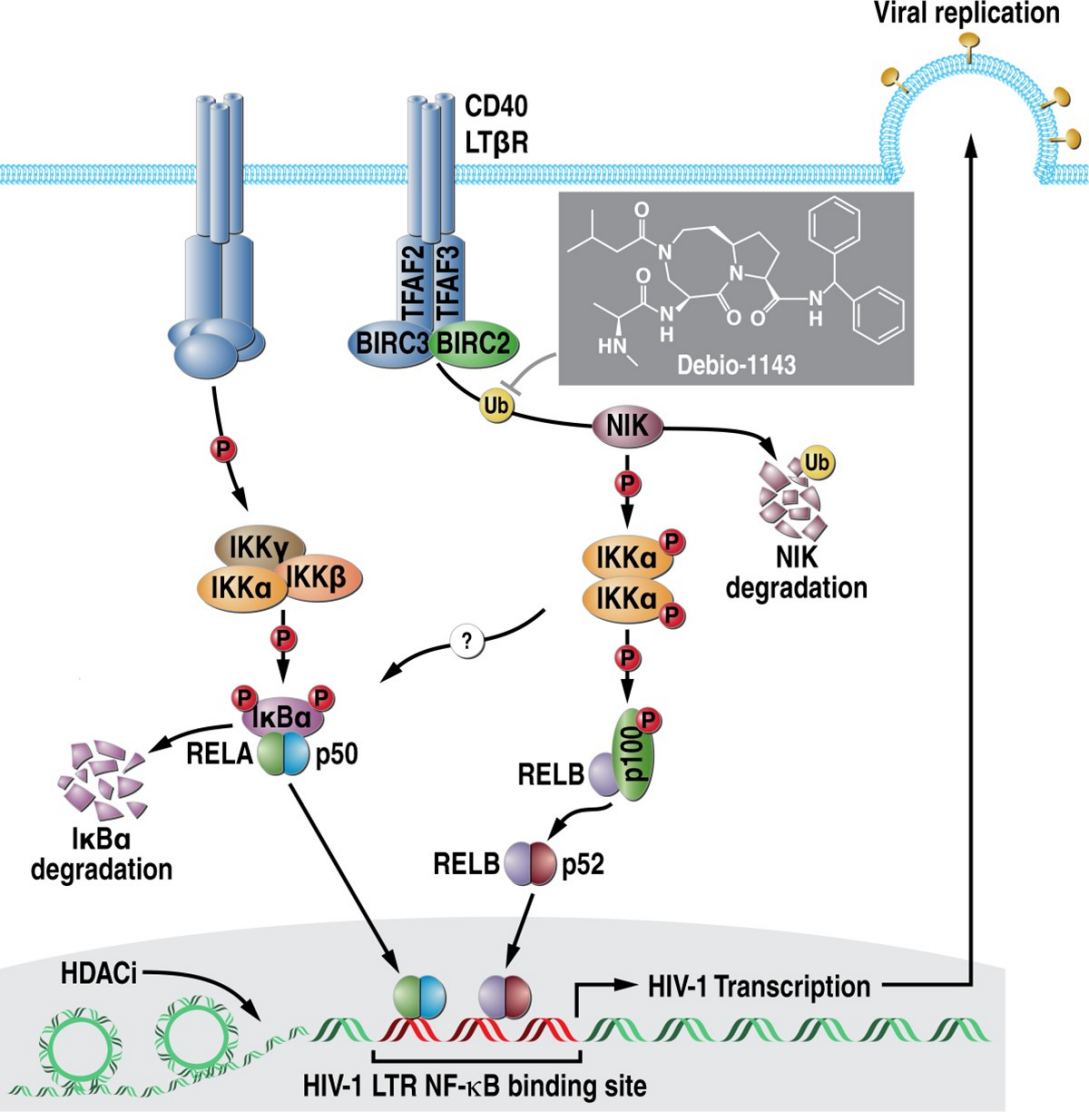
**Model for canonical (left) and non-canonical (right) NF-kB pathways and site of action of Debio 1143** (adapted from Pache *et al*. [33]). **A.** The non-canonical NF-κB pathway occurs through a subset of TNFRs. In the absence of stimulation, a complex of BIRC2, BIRC3 (cIAP2), TRAF2, and TRAF3 degrades NIK. Upon receptor activation, BIRC2 and BIRC3 promote ubiquitination and degradation of TRAF3, permitting an accumulation of NIK. In turn, NIK activation results in the phosphorylation of IKK alpha, leading to the proteolytic processing of p100 into p52. p52 forms a heterodimer with the RELB transcription factor and translocates to the nucleus, inducing the expression of target genes. **B.** Debio 1143 induces the E3 ubiquitin ligase activity of BIRC2, leading to its auto-ubiquitination and degradation.

Canonical and non-canonical NF-kB signaling pathways play an important role in HIV-1 latency reversal, implicating its regulation as an important therapeutic strategy [25–26]. The non-canonical NF-κB pathway occurs through a subset of TNF receptors (TNFRs) (Fig 1). In the absence of stimulation, a complex of BIRC2, BIRC3 (cIAP2), TRAF2, and TRAF3 degrades NF-κB-inducing kinase (NIK). Upon receptor activation, BIRC2 and BIRC3 promote ubiquitination and degradation of TRAF3, permitting an accumulation of NIK. In turn, NIK activation results in the phosphorylation of IkB kinase alpha (IKK alpha), leading to the proteolytic processing of p100 into p52. p52 forms a heterodimer with the RELB transcription factor and translocates to the nucleus, inducing the expression of target genes. Inhibitor of apoptosis protein (IAP) antagonists (IAPa) are small synthetic molecules that mimic a tetrapeptide sequence from second mitochondrial-derived activator of caspases (SMAC). IAPa bind to the baculoviral IAP repeat (BIR) domain that is common to the 8 members of the inhibitor of apoptosis family of proteins, which includes XIAP, BIRC2 (cIAP-1), and BIRC3 (cIAP-2) [27–30]. IAP proteins differ in function, and only BIRC2 and BIRC3 are known modulators of non-canonical NF-κB signaling. IAPa compete with caspases for XIAP binding, but also induces the E3 ubiquitin ligase activity of BIRC2 and BIRC3, leading to auto-ubiquitination and degradation (Fig 1) [31]. Through their ability to bind XIAP, BIRC2 and BIRC3, IAPa elicit pro-apoptotic activities, and have been developed to treat solid and hematological cancers in combination with chemo- and/or radio-therapy [32]. Interestingly this class of agents has also recently emerged as new promising LRAs [33].

In this study, we investigated the potential of IAPa Debio 1143 to reverse HIV-1 latency. We found that Debio 1143 enhances HIV-1 transcription via the NF-kB pathway by degrading the ubiquitin ligase BIRC2, a repressor of the non-canonical NF-kB pathway. We demonstrated that Debio 1143 reverses HIV-1 latency in various HIV-1 latent T cell lines. The combination of Debio 1143 with other LRAs exhibit additive to synergistic effects on HIV-1 latency reversal *in vitro*. We showed that Debio 1143 is one the most efficacious LRA *in vitro*. Importantly, Debio 1143 alone reverses HIV-1 latency in resting CD4+ T cells derived from ART-treated patients. Similarly, Debio 1143 reverses HIV-1 latency in resting CD4+ T cells isolated from HIV-1-infected humanized BLT mice under ART. In conclusion, the IAPa Debio 1143 represents an attractive new LRA candidate, which should be considered to be part of novel pharmacological strategies to eliminate HIV-1 latent cellular reservoirs.

## Materials and methods

### Drugs and antibodies

Debio 1143 was obtained from Debiopharm International S.A., vorinostat, LCL161, TL32711, entinostat and panobinostat from Selleckchem, chaetocin, bryostatin-1, JQ1 and from Sigma, PH02 from ChemBridge, GDC-052 from Santa Cruz Biotechnology, TNF alpha from R&D Systems. Emtricitabine (FTC), tenofovir disoproxil fumarate (TDF) and raltegravir were provided by Drs Baum and Moss. Anti-BIRC2 antibody was obtained from R&D Systems, anti-IkB, anti-p100/p52, RELB, NIK antibodies from Cell Signaling, anti-RELA and anti-CypA antibodies from Santa Cruz Biotechnology, and anti-histone 2B antibody from Bio-Rad.

### HIV-1 infection and LTR activation analyses

CD4+ T-lymphocytes (300,000) derived from peripheral blood mononuclear cells (PBMCs) from blood donors were incubated for 24 h with VSVG-NL4.3-GFP (10 ng of HIV-1 p24 quantified by ELISA (PerkinElmer)) in the presence of absence of the IAPa Debio 1143 and analyzed for infection (GFP content) by FACS. NL4.3-GFP (also called pHIV-GFP) was provided by Drs. C. Aiken and D. Gabuzda. NL4.3-GFP encodes full-length NL4.3 proviral DNA and expresses GFP in place of *nef* [34]. NF-κB reporter (Luc)-3T3 cells (BPS Bioscience) (100,000) (triplicate) were treated with Debio 1143 at the indicated concentrations and luciferase activity in cell lysates was quantified after 6 h.

### *In vitro* HIV-1 latency reversal analysis in HIV-1 latent cell lines

Latently HIV-1 infected JLat 10.6, 2D10 and 5A8 GFP reporter cells [35–37] (250,000) were treated with the indicated compounds at the indicated concentrations for 48 h and latency reversal was quantified by FACS for GFP expression.

### *Ex vivo* HIV-1 latency reversal analysis in resting CD4+ T cells derived from ART-treated patients

Human resting CD4+ T cells were isolated from PBMCs derived from ART-treated HIV-1-infected patients using the EasySep Human Resting CD4+ T Cell Isolation Kit (immunomagnetic negative selection). Isolated resting CD4+ T cells were first serially diluted and seeded into wells. Each dilution was then treated with compounds as indicated for two days, MOLT-4-CCR5 cells [38] were then added to each dilution to propagate released virions. MOLT-4-CCR5 cells and released virions were spinoculated in order to increase levels of infections as described previously [39]. Supernatants were collected at day 7 and split in two for i) HIV-1 RNA quantification by RT-qPCR as we described previously [40–41] and for ii) HIV-1 infection after spinoculation on TZM indicator cells. TZM infection was quantified by measuring β-galactosidase activity levels in cell lysates. Note that PBMCs were obtained directly from patients by the authors. The UCSD Human Research Protections Program (Institutional Review Board) approved the study protocol, consent and all study related procedures. All study participants provided voluntary, written informed consent before any study procedures were undertaken.

### BIRC2 degradation and transcription factors subcellular analyses

CD4+ T-lymphocytes (1,000,000) were treated with the indicated compounds for the indicated periods of time and analyzed for the expression of various host proteins including components of the NF-kB signaling pathway by Western blotting. For the analysis of the subcellular localization (nuclear versus cytosolic) of selected HIV-1 transcription factors, NE-PER Nuclear and Cytoplasmic Extraction kit from ThermoScientific Fisher (Pierce) was used according to the manufacturer’s instructions. siRNA control and siRNA BIRC2 were obtained from Sigma and used according to the manufacturer’s instructions.

### RELA and RELB association with HIV-1 LTR analysis

2D10 cells (500,000) (were treated with 1 μM Debio 1143 for 10 h prior to ChIP analysis using control, anti-RELB or anti-RELA antibodies as described previously [33]. RELB- and RELA-specific association with the HIV-1 LTR and the IkB alpha gene promoter region, or an intergenic region upstream of the PABPC1 gene not known to contain NF-kB binding sites as negative control, was analyzed by qPCR as described previously [33].

### Cellular toxicity analyses

CD4+ T-lymphocytes were exposed to the indicated compounds at the indicated concentrations for 24 h. Cytotoxicity of compounds was analyzed by Lactate Dehydrogenase (LDH) Assay (Cayman Chemical). LDH release from cells into the culture supernatant was quantified according to the manufacturer’s instructions. Absorbance was measured at 490/690 nm using a microplate spectrophotometer.

### Cytokine and chemokine analysis in mice

PBMCs from an ART-treated patient were incubated with DMSO, anti-CD3/CD28 mAbs or Debio 1143 at the indicated concentrations. Supernatants were collected after 24 h, and cytokine concentrations were quantified by BioPlex analysis. Bio-Plex Pro Assays are immunoassays formatted on magnetic beads that utilize principles similar to those of a sandwich ELISA. Capture antibodies against the biomarker of interest are covalently coupled to the beads. A biotinylated detection antibody creates the sandwich complex, and the final detection complex is formed by the addition of a streptavidin-phycoerythrin (SA-PE) conjugate, where PE serves as the fluorescent reporter. Reactions are read using a Luminex-based reader. Cytokine mRNAs levels in PBMCs were quantified by PCR using β-actin as control. Blood from HIV-1-infected and ART-treated humanized BLT mice, which received either vehicle or Debio 1143 (100 mg/kg, p.o.), was collected 48 h post-IAPa administration, and human cytokine levels were quantified by BioPlex analysis.

### Generation of humanized BLT mice

Humanized BLT mice were generated as described previously [40–45], by implanting 1-mm^3^ pieces of human fetal liver and thymus tissues (Advanced Bioscience Resources) under the kidney capsule in 6 to 8-week-old female NSG mice (Jackson Laboratories) bred at The Scripps Research Institute (TSRI). Each cohort was produced with tissues from a single donor. CD34+ HSPC were purified from autologous fetal liver tissue, isolated by magnetic bead selection for CD34+ cells (Miltenyi), phenotyped cytometrically [40–45], and cryopreserved until injection (200,000–350,000 CD34+ cells) into mice 3 weeks after Thy/Liv implantation. Human reconstitution in peripheral blood was verified by flow cytometry as described previously [40–45]. Mice were maintained at the Department of Animal Resources (DAR) at TSRI in accordance with protocols approved by the TSRI Ethics Committee, the Institutional Animal Care and Use Committee (Permit Number: 13–0001). This study was carried out in strict accordance with the recommendations in the Guide for the Care and Use of Laboratory Animals of the National Institutes of Health. All surgery was performed under sodium pentobarbital anesthesia, and all efforts were made to minimize suffering. The method of sacrifice used for the experimental mice is cervical dislocation. A power calculation was used to determine the sample size (number of mice/group).

### HIV-1 infection of humanized BLT mice

Stocks of HIV JR-CSF [46–47] were prepared as previously described [40–45] and standardized by p24 ELISA. Humanized BLT mice were challenged i.v. with HIV-1 (100 ng of p24). Three weeks HIV-1 post-challenge, infection was confirmed by quantifying viral RNA by PCR viral load in peripheral blood (plasma) using one-step reverse transcriptase quantitative real-time PCR (ABI custom TaqMan Assays-by-Design) according to the manufacturer’s instructions. Primers were 5-CATGTTTTCAGCATTATCAGAAGGA-3 and 5-TGCTTGATGTCCCCCCACT-3, and MGB-probe 5-FAM-CCACCCCACAAGATTTAAACACCATGCTAA-Q-3, where FAM is 6-carboxyfluorescein as we recently described [40–41]. The assay sensitivity was of 423 RNA copies per mL.

### *Ex vivo* HIV-1 latency reversal analysis in resting CD4+ T cells derived from ART-treated HIV-1-infected humanized BLT mice

Human resting CD4+ T cells were isolated from blood, thymic organoid, lung, spleen, bone marrow, lymph nodes and liver of HIV-1-infected BLT mice under ART. Isolated human resting CD4+ T cells were pooled, counted, split in triplicate and treated with vehicle, PMA (20 ng/mL) + ionomycin (1 μg/mL), Debio 1143 (1 μM), LCL161 (1 μM), panobinostat (1 μM) or vorinostat (1 μM). *De novo* released virions from supernatants were purified, concentrated and quantified by RT-qPCR.

## Results

### The inhibitory apoptosis protein antagonist (IAPa) Debio 1143 enhances HIV-1 transcription via NF-kB signaling

**Fig 2:**
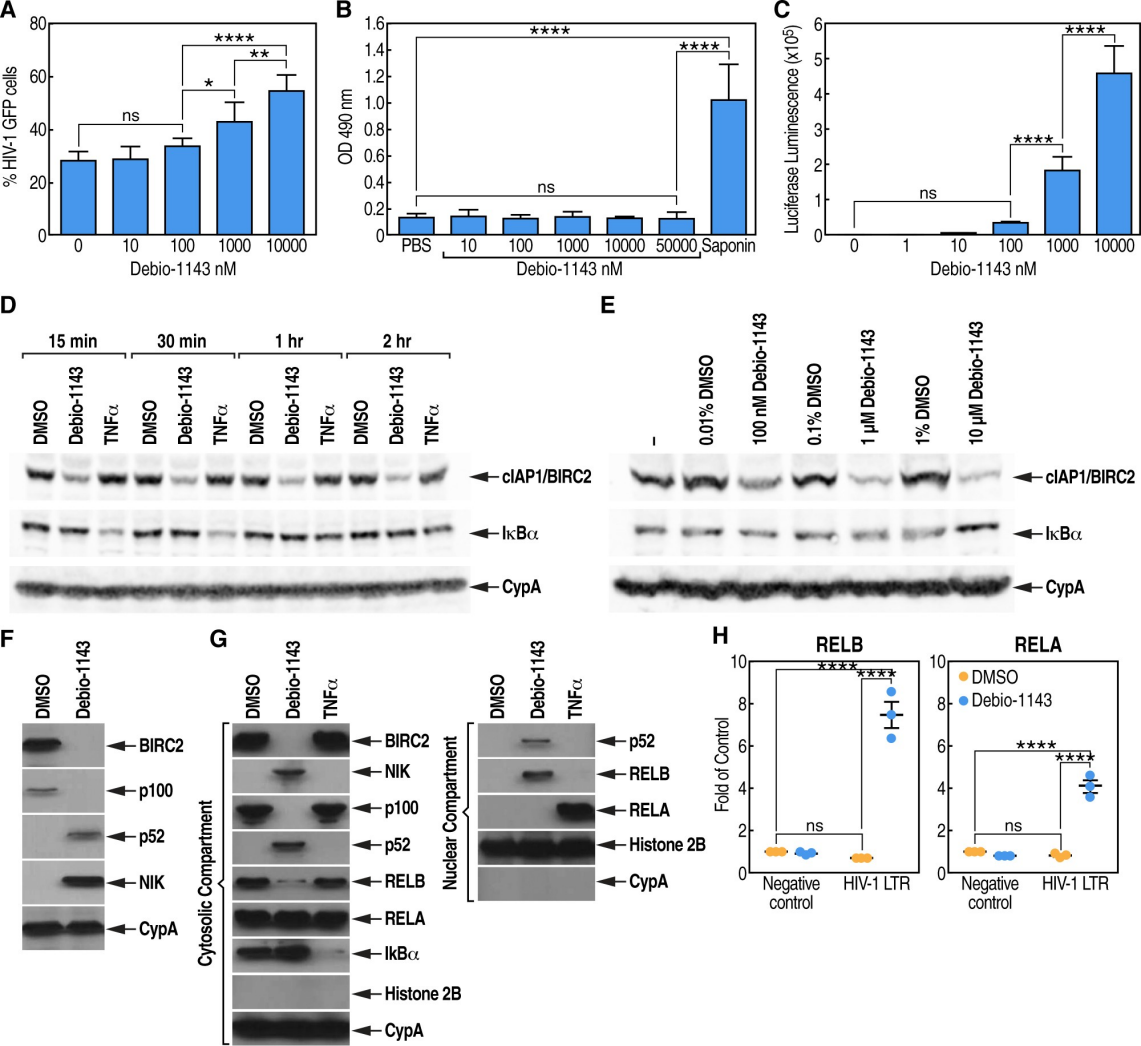
Analysis of the mechanisms of action of Debio 1143. CD4+ T-lymphocytes (100,000) (triplicate) were incubated with VSVG-NL4.3-GFP and Debio 1143 (0 to 10 μM) for 24 h and analyzed for infection by FACS (**A**) and for cytotoxicity by LDH assay (**B**). **C.** NF-κB reporter (Luc)-3T3 cells (BPS Bioscience) (100,000) (triplicate) were treated with Debio 1143 and luciferase activity in cell lysates was quantified after 6 h. **D.** CD4+ T-lymphocytes (1,000,000) were treated (from 15 min to 2 h) with DMSO, Debio 1143 (1 μM) and TNF alpha (10 ng/mL) and cell lysates analyzed by Western blotting for BIRC2, IkB alpha and CypA expression. **E.** Same as **D** except that cells were treated for 15 min with 0, 0.1, 1 and 10 μM of Debio 1143. **F.** Same as **D** except that cells were treated for 24 h with DMSO or 1 μM of Debio 1143, and cell lysates analyzed by Western blotting for BIRC2, p100/52, NIK and CypA expression. **G.** Same as **D** except that cells were treated for 24 h with DMSO, 1 μM of Debio 1143 or 10 ng/mL of TNF alpha, and cytosolic and nuclear extracts analyzed by Western blotting for the expression of various components of the NF-kB signaling pathways and cytosolic and nuclear markers. **H.** 2D10 cells (500,000) (triplicate) were treated with 1 μM Debio 1143 for 10 h prior to ChIP analysis using control, anti-RELB or anti-RELA IgG. RELB- and RELA-specific association with the HIV-1 LTR and the IkB alpha gene promoter region, or an intergenic region upstream of the PABPC1 gene not known to contain NF-kB binding sites as negative control, was analyzed by qPCR and is presented as fold enrichment over control IgG. Data from **A** to **F** are each representative of two independent experiments. P-values are presented. ANOVA and Bonferroni’s multiple comparison tests were used to compare means of different groups. Bar graphs indicate each sample point, mean, and SEM. Key for adjusted p-values from Bonferroni’s multiple comparison tests on graphs: ns = no significance, * = 0.01≤p<0.05, ** = 0.001 ≤p<0.01, *** = 0.0001≤p<0.001, and **** = p<0.0001.

The HIV-1 LTR contains two copies of an NF-kB enhancer element recognized by the canonical RELA:p50 heterodimer [25] or the non-canonical RELB:p52 heterodimer [48–49]. Importantly, IAPa have recently emerged as promising LRAs [33]. Since BIRC2 (cIAP1) is both a positive regulator of canonical and a negative regulator of the non-canonical NF-kB signaling pathways and that the IAPa Debio 1143 binds with high affinity to BIRC2 (5.1 nM) [50], we asked whether Debio 1143 could serve as LRA by acting on NF-kB signaling to promote HIV-1 transcription. To test this hypothesis, we examined whether Debio 1143 enhances HIV-1 infection. Specifically, CD4+ T cells were incubated for 24 h with Debio 1143 together HIV-1-GFP and levels of infection were quantified by FACS. We also analyzed the cellular toxicity of Debio 1143 by lactate dehydrogenase (LDH) assay, which is a reliable colorimetric assay to quantitatively measure LDH released into the media from damaged cells as a biomarker for cellular cytotoxicity and cytolysis. We found that Debio 1143 enhanced in a dose-dependent manner HIV-1 infection at clinically relevant concentrations (Fig 2A). Importantly, at high concentrations, Debio 1143 did not exert any cytotoxicity (Fig 2B).

We then examined whether Debio 1143 activates NF-kB signaling directly. We took advantage of the NF-κB reporter (Luc)-3T3 cell line, which is designed to monitor NF-κB signal transduction pathways. It contains a luciferase gene driven by four copies of the NF-κB response element. After activation by pro-inflammatory cytokines or stimulants of lymphokine receptors, endogenous NF-kB transcription factors bind to the DNA response elements, inducing transcription of the luciferase reporter gene. NF-κB reporter (Luc)-3T3 cells were treated with Debio 1143 and luciferase activity in cell lysates was quantified after 6 h. Importantly, Debio 1143 triggered activation of the NF-kB signaling (Fig 2C), suggesting that Debio 1143 possesses the ability to activate the HIV-1 LTR by acting on the two copies of the NF-kB enhancer element.

### Debio 1143 triggers rapid degradation of BIRC2 in CD4+ T lymphocytes

We then asked whether Debio 1143 activates the HIV-1 LTR by acting on the non-canonical NF-kB signaling since the IAPa binds with high affinity to the negative regulator of the non-canonical NF-kB signaling—BIRC2 [50]. CD4+ T cells were treated with DMSO, Debio 1143 and TNF alpha and analyzed for BIRC2 expression. Debio 1143, but not TNF alpha, triggers rapid degradation of BIRC2 (Fig 2D). TNF alpha, triggers IkB alpha degradation with a rebound after 1 h, while Debio 1143 has no effect. This is in accordance with the notion that IkB alpha is a hallmark of canonical NF-kB signaling activation. Similar levels of cyclophilin A (CypA) indicate that similar amounts of cell lysates were analyzed. We also found that the degradation of cellular BIRC2 by Debio 1143 is dose-dependent (Fig 2E). Together these data suggest that the IAPa Debio 1143, by binding to BIRC2 with high affinity [50], triggers the E3 ubiquitin ligase activity of BIRC2, leading to its auto-ubiquitination and degradation (Fig 1) [31].

According to the model of the activation of the non-canonical NF-kB signaling pathway (Fig 1), we found that Debio 1143-mediated BIRC2 degradation results into an accumulation of NIK, and the proteolytic processing of p100 into p52 (Fig 2F). As above, similar CypA levels indicate that similar amounts of cell lysates were analyzed. To further understand the mechanisms of action of the Debio 1143-mediated activation of the non-canonical NF-kB signaling pathway that leads to the LTR-dependent transcriptional activation of HIV-1, we examined the nuclear translocation of NF-kB transcription factors triggered by the treatment of CD4+ T cells by Debio 1143. We found that p52 and RELB translocated into the nuclear compartment while RELA did not (Fig 2G). In contrast, the activation of the canonical NF-kB signaling pathway by TNF alpha induces RELA nuclear translocation (Fig 2G). CypA and histone 2B served as cytosolic and nuclear markers, respectively. The efficient nuclear translocation of RELB and p52 upon Debio 1143 further indicates that the IAPa stimulates HIV-1 transcription via activation of the non-canonical NF-kB signaling pathway.

### Debio 1143 mediates RELB association with HIV-1 LTR

The rapid Debio 1143-mediated degradation of BIRC2 and p52/RELB nuclear translocation suggests that the IAPa modulates HIV-1 transcription by acting on the non-canonical NF-kB pathway. To test this hypothesis, we examined by chromatin immunoprecipitation (ChIP) whether Debio 1143 via stimulation of the non-canonical signaling modulates the binding of NF-kB transcription factors to the HIV-1 LTR. Importantly, we found that Debio 1143 triggers the association of RELB with the HIV-1 LTR (Fig 2H), further suggesting the participation of non-canonical transcription factors such as RELB in the reactivation of HIV-1 transcription. Although at a lesser degree, Debio 1143 also triggered an association of RELA with the HIV-1 LTR (Fig 2H), suggesting a concerted action of both canonical and non-canonical transcription factors in HIV-1 transcription reactivation by the IAPa. These findings suggest that Debio 1143 mainly triggers the non-canonical NF-kB pathway by activating the RELB:p52 heterodimer for HIV-1 LTR binding and activation of LTR-dependent transcription (Fig 1). This is in accordance with the fact that cellular IAPs such as BIRC2 are also important in the canonical NF-kB signaling activation [33].

### Debio 1143 is a potent LRA in HIV-1 latent cells

**Fig 3:**
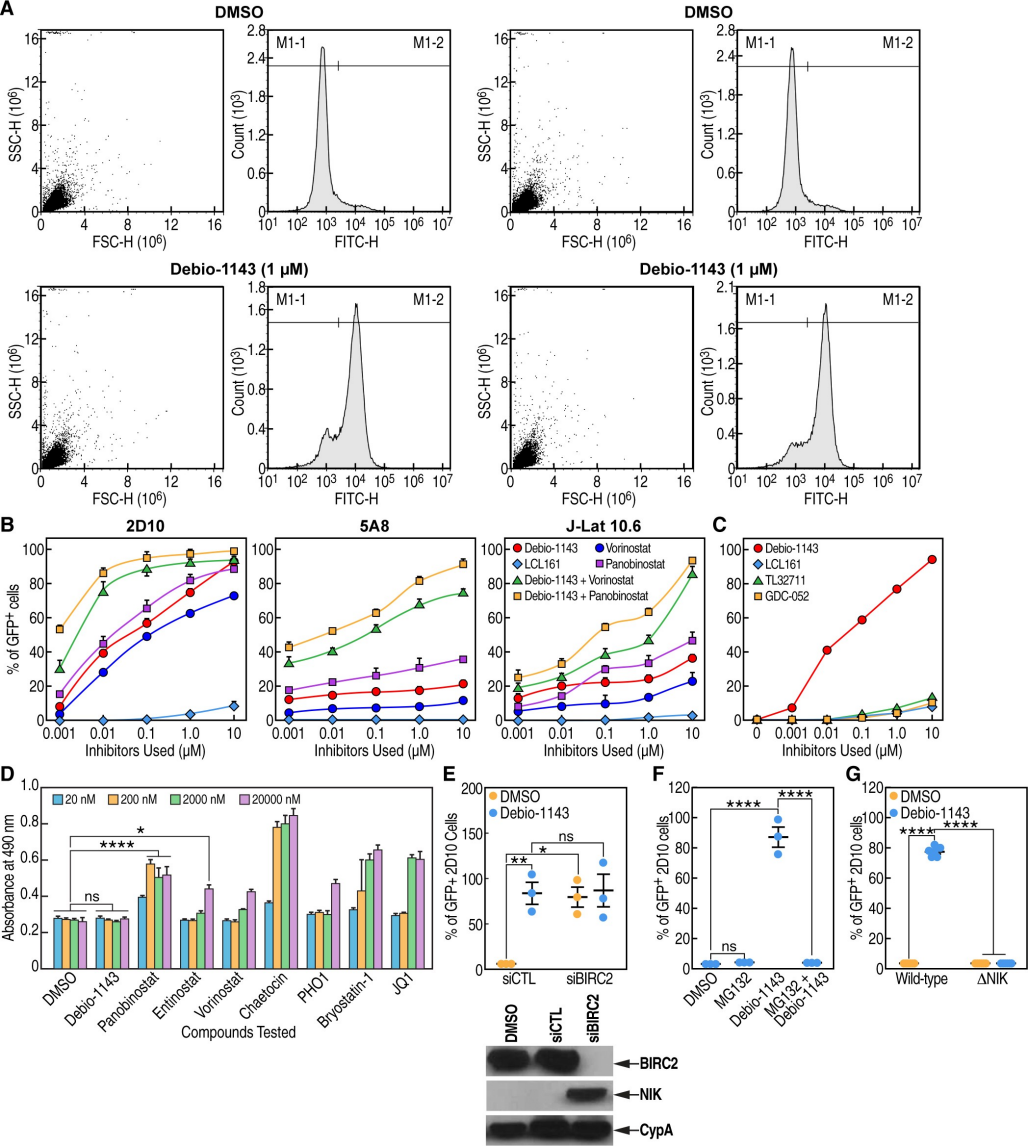
Debio 1143 reverses HIV-1 latency. **A**. Typical FACS graphs for the percentage of GFP+ HIV-1 latent reporter cells. The population on the forward scatter/side scatter dot plot was placed by the automatic scaling feature of the cytometer. Minor variability between reporter cell replicates was observed as shown by representative graphs of 2D8 cells treated with control DMSO or Debio 1143 (1 μM). **B.** Latently infected JLat 10.6, 2D10 and 5A8 GFP reporter cells (250,000) (duplicate) were treated for 48 h with LCL161 (1 μM), Debio 1143 (1 μM) or in combination with panobinostat (10 nM) and vorinostat (500 nM). GFP expression was quantified by FACS. Data are expressed as % of GFP levels. Two distinct experiments were conducted in triplicate and averaged data are presented. **C.** Same as **B** except that the capacity of Debio 1143 at reversing HIV-1 latency in 2D10 cells was compared with that of other IAPa. **D.** CD4+ T-lymphocytes (250,000) (triplicate) were incubated with the LRAs and cytotoxicity was quantified after 48 h by LDH assay as above (Fig 2). **E.** 2D10 cells were treated with control (siCTL) and BIRC2 (siBIRC2) siRNA for 24 h, then incubated with DMSO or Debio 1143 (1 μM) and analyzed for GFP expression after 48 h (left panel) and cell lysates analyzed by Western blotting for BIRC2, NIK and CypA expression. **F.** 2D10 cells (triplicate) were first incubated with or without MG132 (25 μM) for 30 min, subsequently exposed to DMSO or Debio 1143 (1 μM) for 48 h, and analyzed by FACS for GFP content. **G.** Wild-type or NIK knockout (ΔNIK) 2D10 cells were exposed to DMSO or Debio 1143 (1 μM) for 48 h, and analyzed by FACS for GFP content. Two distinct experiments were conducted in triplicate and averaged data are presented. P-values are presented. ANOVA and Bonferroni’s multiple comparison tests were used to compare means of different groups. Bar graphs indicate each sample point, mean, and SEM. Key for adjusted p-values from Bonferroni’s multiple comparison tests on graphs: ns = no significance, * = 0.01≤p<0.05, ** = 0.001 ≤p<0.01, *** = 0.0001≤p<0.001, and **** = p<0.0001.

We then investigated the possibility that Debio 1143, by promoting HIV-1 transcription via BIRC2 degradation and activation of the non-canonical and canonical NF-kB signaling pathways, possesses the ability of reversing viral latency in CD4+ T cells. We used latently HIV-1-infected CD4+ JLat 10.6, 2D10 and 5A8 reporter cell lines that contain an integrated viral genome with a GFP gene [35–37]. Reporter HIV-1 latent cells were treated with increasing concentrations of Debio 1143 and latency reversal was quantified by GFP expression. We tested another IAPa—LCL161 –which is currently clinically tested for the treatment of patients with solid and hematological cancers [51–52]. We also tested Debio 1143 in combination with other LRAs, two HDAC inhibitors—panobinostat and vorinostat [13–14]. Remarkably, Debio 1143 efficiently reversed HIV-1 latency in the three HIV-1 latent CD4+ T cell lines (Fig 3B). Note that we did not use any gating on the cells (Fig 3A). The percentage of GFP is from the total population and 85–90% of cells were viable. The population on the forward scatter/side scatter dot plot was placed by the automatic scaling feature of the cytometer, and in general, the cell population looked perfectly uniform. We observed minor variability between replicates for reporter cells as shown by representative cell graphs in Fig 3A. When combined with panobinostat and vorinostat, the latency reversal activity of Debio 1143 was amplified (Fig 3B). Debio 1143 was as efficient or even more efficient than other IAPa tested such as LCL161 [53], TL32711 (Birinapant) [54] and GDC-052 [55] (Fig 3C). In contrast to Debio 1143, several LRAs were toxic to cells (Fig 3D). We obtained similar data with JLat 10.6 and 5A8 cells (data not shown). Thus, the IAPa Debio 1143 represents a highly potent IAPa with limited cytotoxic properties *in vitro*.

Since we showed above that Debio 1143 triggers BIRC2 degradation (Fig 2) and reverses HIV-1 latency (Fig 3B and 3C), we asked whether suppressing expression of BIRC2 by siRNA would also reverse HIV-1 latency. Treating 2D10 cells with siRNA targeting BIRC2, but not with siRNA control, not only triggered degradation of BIRC2 and accumulation of NIK, it also reversed HIV-1 latency at levels similar to those of Debio 1143 (Fig 3E), further suggesting that BIRC2 represents a main target of Debio 1143.

Previous work suggested that IAPa induce the E3 ubiquitin ligase activity of BIRC2, leading to its auto-ubiquitination and degradation [31]. We thus asked whether neutralizing the proteasome machinery would prevent the Debio 1143-mediated HIV-1 latency reversal. To test this hypothesis, latently HIV-1-infected 2D10 cells were first incubated with MG132 for 30 min, subsequently exposed to Debio 1143 for 48 h, and analyzed by FACS for efficiency of HIV-1 latency reversal. Importantly, we found that MG132 treatment totally inhibited HIV-1 latency reversal by Debio 1143 (Fig 3F). This data further indicates that the degradation of BIRC2 by Debio 1143 controls HIV-1 latency reversal.

As mentioned above, in the absence of stimulus, an intracellular protein complex composed of BIRC2 (cIAP1), BIRC3 (cIAP2), TRAF2 and TRAF3 degrades the NF-κB-inducing kinase (NIK), keeping to the non-canonical NF-kB signaling silent (Fig 1). Consistent with this notion, we found that BIRC2 degradation by Debio 1143 stabilizes NIK expression (Figs 2 and 3), and reverses HIV-1 latency (Figs 2 and 3). We then asked whether the suppression of NIK expression would prevent the Debio 1143-mediated HIV-1 latency reversal. To test this hypothesis, we took advantage of a NIK knockout 2D10 cell line created by the Chanda lab [33]. In contrast to parental 2D10 cells, we found that Debio 1143 was unable to reverse HIV-1 latency in NIK knockout 2D10 cells (Fig 3F), further supporting the notion that the lack of NIK expression prevents the activation of the non-canonical NF-kB signaling pathways by Debio 1143 (Fig 1).

### Debio 1143 reverses HIV-1 latency in resting CD4+ T cells isolated from ART-treated patients

After demonstrating that Debio 1143 reverses HIV-1 latency in latently viral-infected CD4+ T cell lines (Fig 3), we examined whether the IAPa mediates a similar effect in a more physiological context–in resting CD4+ T cells isolated from PBMCs derived from ART-treated HIV-1 patients. In patients under ART, a majority of proviruses are defective [56]. Thus, we used two distinct but complementary assays to quantify the degree of HIV-1 latency reversal in isolated resting CD4+ T cells: i) quantification of virion HIV-1 RNA in culture supernatant by RT-qPCR with single copy sensitivity as described previously [56] that overestimates amounts of released virions after LRA treatment due to the presence of defective virions; and ii) quantification of infectious virions using the quantitative viral outgrowth assay (QVOA) as described previously [57].

**Fig 4:**
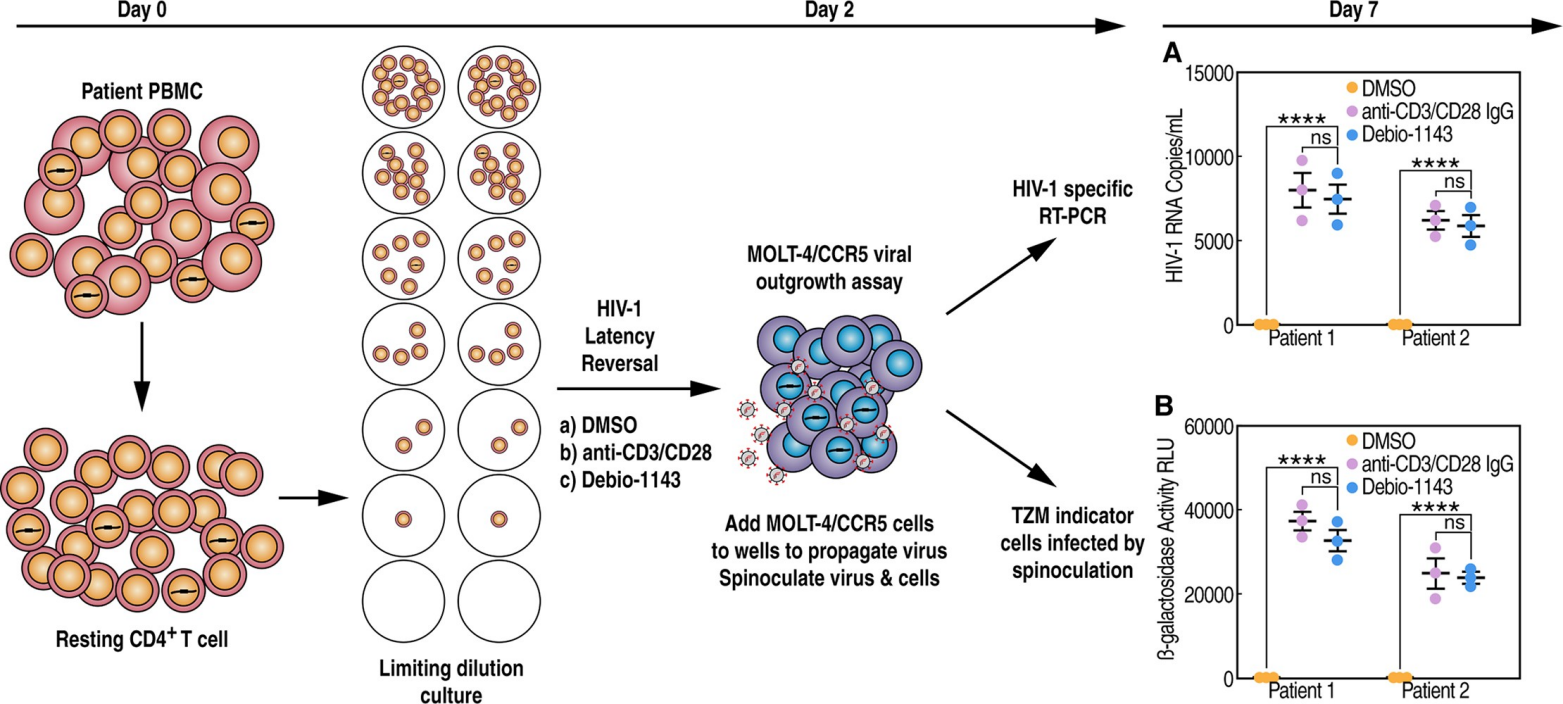
Debio 1143 reverses HIV-1 latency in resting CD4+ T cells from ART-treated patients. Purified resting CD4+ T cells from two ART-treated patients were serially diluted fivefold from 1,000,000 cells per well to 1,600 cells per well and seeded into individual wells of 48-well plates (5 replicates at each dilution). Each dilution was then treated with DMSO, anti-CD3/CD28 IgG-coated microbeads or 1 μM of Debio 1143. At day 2, highly permissive MOLT-4-CCR5 cells were added to each dilution to propagate released virions after latency reversal by LRAs. The ratio of target cells added was 10^7^ MOLT-4-CCR5 cells to the 1x10^6^ resting CD4+ T cell dilution, and 10^6^ MOLT-4-CCR5 cells to all other resting CD4+ T cell dilutions. To increase infection, MOLT-4-CCR5 cells and released virions were spinoculated. Supernatants were collected at **day 7** and split for HIV-1 RNA quantification (**A**) and HIV-1 infection (**B**) in triplicate. HIV-1 RNA was measured by RT-qPCR. Control experiments showed that >95% of HIV-1 RNA in supernatants could be pelleted by centrifugation at 24,000 g for 1 h and were resistant to DNase I treatment. Infectivity of released virions was scored using TZM indicator cells after spinoculation. TZM infection was scored after 48 h by β-galactosidase activity in cell lysates. P-values are presented. ANOVA and Bonferroni’s multiple comparison tests were used to compare means of different groups. Bar graphs indicate each sample point, mean, and SEM. Key for adjusted p-values from Bonferroni’s multiple comparison tests on graphs: ns = no significance, * = 0.01≤p<0.05, ** = 0.001 ≤p<0.01, *** = 0.0001≤p<0.001, and **** = p<0.0001.

Resting CD4+ T cells were isolated from a pool of frozen PBMCs derived from ART-treated HIV-1-infected patients by immunomagnetic negative selection. The EasySe procedure involves labeling unwanted cells with antibody complexes and magnetic particles. The magnetically labeled cells are separated from the untouched desired cells by an EasySep magnet. First, purified resting CD4+ T cells were serially diluted fivefold from 1,000,000 cells per well to 1600 cells per well and seeded into wells of 48-well plates (5 replicates at each dilution) (Fig 4). Second, each dilution was treated with DMSO, anti-CD3/CD28 IgG-coated microbeads or 1 μM of Debio 1143. At day 2, highly permissive MOLT-4-CCR5 cells were added to each dilution to propagate released virions after latency reversal by LRAs as described previously [58]. The ratio of target cells added was 10^7^ MOLT-4-CCR5 cells to the 1x10^6^ resting CD4+ T cells dilution, and 10^6^ MOLT-4-CCR5 cells to all other resting CD4+ T cells dilutions. To increase infection, MOLT-4-CCR5 cells and released virions were spinoculated as described previously [59].

Supernatants were collected at day 7 and split for i) HIV-1 RNA quantification; and ii) HIV-1 infection. HIV-1 RNA was measured by RT-qPCR. Control experiments showed that >95% of HIV-1 RNA in supernatants could be pelleted by centrifugation at 25,000 g for 1 h and were resistant to DNase I treatment. Infectivity of released virions was scored using TZM indicator cells after spinoculation of virions onto TZM cells. We found that the spinoculations enhanced the sensitivity of the QVOA (9-13-fold). Debio 1143 enhanced HIV-1 RNA levels (Fig 4A) and TZM infection (Fig 4B), further suggesting that Debio 1143 is a potent LRA. By comparing TZM infection levels and p24 levels in supernatants, we found that the Debio 1143-mediated increase of infectivity of virions was reduced compared to that of the amounts of released virions (~50-fold versus 500-5000-fold), reflecting and confirming a significant percentage of defective virions in the supernatant detected by RT-qPCR (Fig 4). Thus, Debio 1143 possesses the ability to reverse *ex vivo* HIV-1 latency in resting CD4+ T cells derived from ART-treated HIV-1 patients.

### Debio 1143 reverses HIV-1 latency in resting human CD4+ T cells isolated from ART-treated humanized BLT mice

**Fig 5:**
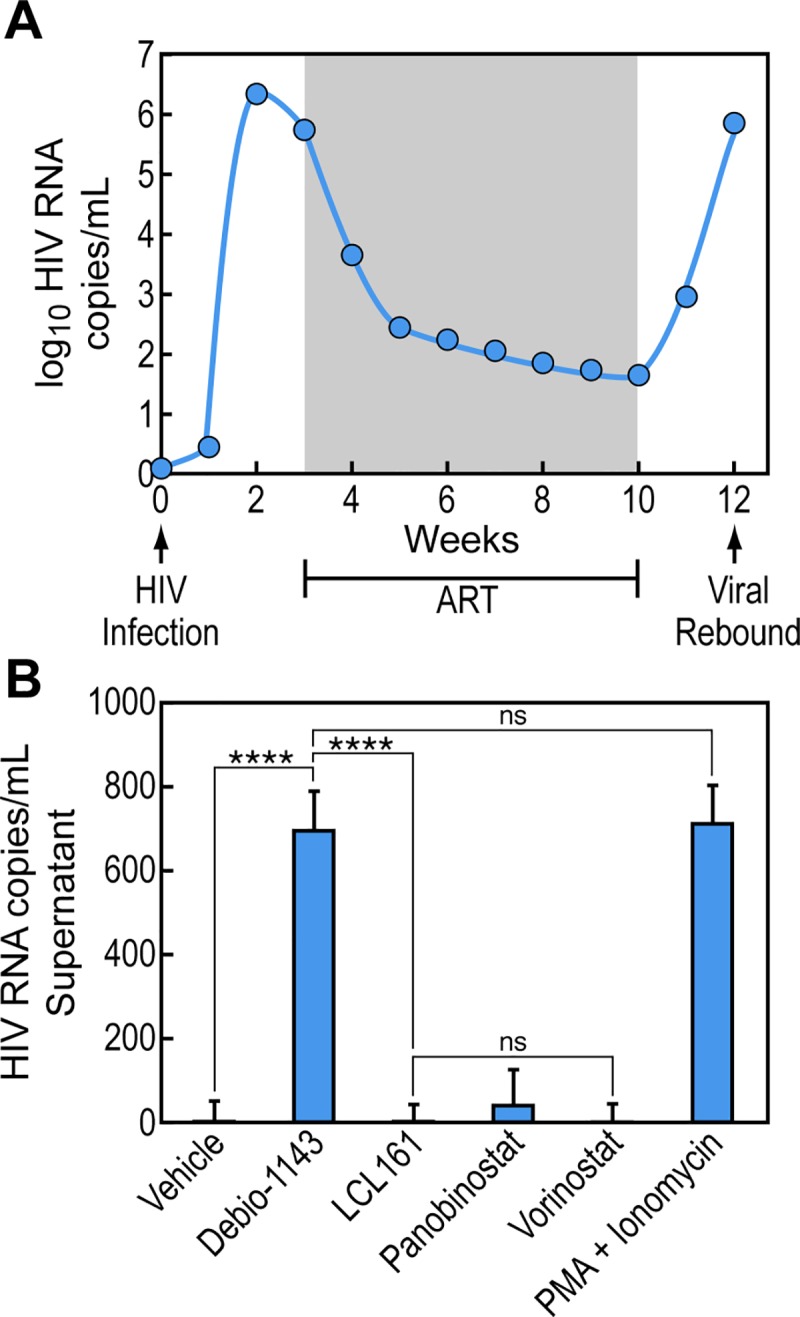
Debio 1143 reverses *ex vivo* HIV-1 latency in resting CD4+ T cells isolated from BLT mice. **A.** Experimental design for the HIV-1 latency model in BLT mice. When ART is interrupted, viral rebound occurs **B.** Same as **A** except that ART was maintained until isolation of resting CD4+ T cells from blood, thymic organoid, lung, spleen, bone marrow, lymph nodes and liver of HIV-1-infected BLT mice (5 mice per treatment). Isolated resting CD4+ T cells from each group were pooled, counted, split in triplicate and treated with vehicle, PMA (20 ng/mL) + ionomycin (1 μg/mL), Debio 1143 (1 μM), LCL161 (1 μM), panobinostat (1 μM) or vorinostat (1 μM). *De novo* released virions from supernatants were purified as above and quantified by RT-qPCR. Data are expressed in copies of HIV-1 RNA/mL of supernatant. P-values are presented. ANOVA and Bonferroni’s multiple comparison tests were used to compare means of different groups. Bar graphs indicate each sample point, mean, and SEM. Key for adjusted p-values from Bonferroni’s multiple comparison tests on graphs: ns = no significance, * = 0.01≤p<0.05, ** = 0.001 ≤p<0.01, *** = 0.0001≤p<0.001, and **** = p<0.0001.

We recently used the humanized BLT mouse model to test the microbicidal efficacy of ARTs [40] and a viral membrane-disrupting peptide C5A [41], which we previously identified to be a potent anti-HIV-1 agent *in vitro* [60] and in macaques [61]. The Garcia [62] and Zack labs [63] demonstrated that HIV-1 latency can be developed in BLT mice. We thus investigated the capacity of Debio 1143 to reverse HIV-1 latency in ART-treated BLT mice (Fig 5A). Briefly, BLT mice were infected with JR-CSF (200 ng of p24), after 3 weeks, infection was verified by qPCR as we described previously [38–39]. Mice were then treated daily with ART (FTC 150 mg/kg + TDF 150 mg/kg + Raltegravir 80 mg/kg) for 7 weeks (week 3 to 10). Three mice were sacrificed to verify by qPCR that HIV-1 RNA levels were drastically reduced not only in the circulation, but also in tissues (data not shown). At week 10, ART was interrupted and viral rebound was observed at week 12 (Fig 5A), indicating that an HIV-1 latency model in BLT mice mimics conditions similar to those observed in ART-treated HIV-1-infected patients.

Other groups of BLT mice were infected with HIV-1 and treated with ART as above, but at week 10 (after 7 weeks of ART), mononuclear cells were isolated from blood, bone marrow, spleen, lymph nodes, and lung as described previously [24, 62]. Isolated mononuclear cells of each mouse and of each tissue from the group of 10 mice were pooled for human resting CD4+ T cells isolation as described previously [24, 62–63] (Easysep Mouse/Human Chimera Isolation Kits). This purification leads to >95% of human resting CD4+ T cells defined by CD4, CCR7 and CD27 expression and lack of expression of CD8, CD11b, CD25 and HLA-DR. We obtained 4–6 million human resting CD4+ T cells per mouse. Resting CD4+ T cells were then treated *ex vivo* with Debio 1143, LCL161, panobinostat and vorinostat for 48 h and amounts of HIV-1 RNA in supernatants were quantified by RT-qPCR (Fig 3). We did not use the QVOA since human resting CD4+ T cell numbers were too low to produce enough infectious virions after LRA treatment after MOLT-4-CCR5 propagation and TZM infection. The *ex vivo* Debio 1143 treatment of human resting CD4+ T cells isolated from blood and tissues reversed HIV-1 latency as demonstrated by high viral RNA levels compared to those of DMSO treatment (Fig 5B). Panobinostat partially reversed HIV-1 latency, but not LCL161 and vorinostat (Fig 5B). Thus, Debio 1143 reverses *ex vivo* HIV-1 latency in human resting CD4+ T cells isolated from HIV-1-infected BLT mice under ART. Variations between individual responses within a mouse group (treatment) to Debio 1143 were surprisingly small.

### Debio 1143 does not stimulate the production of pro-inflammatory cytokines in BLT mice

**Fig 6:**
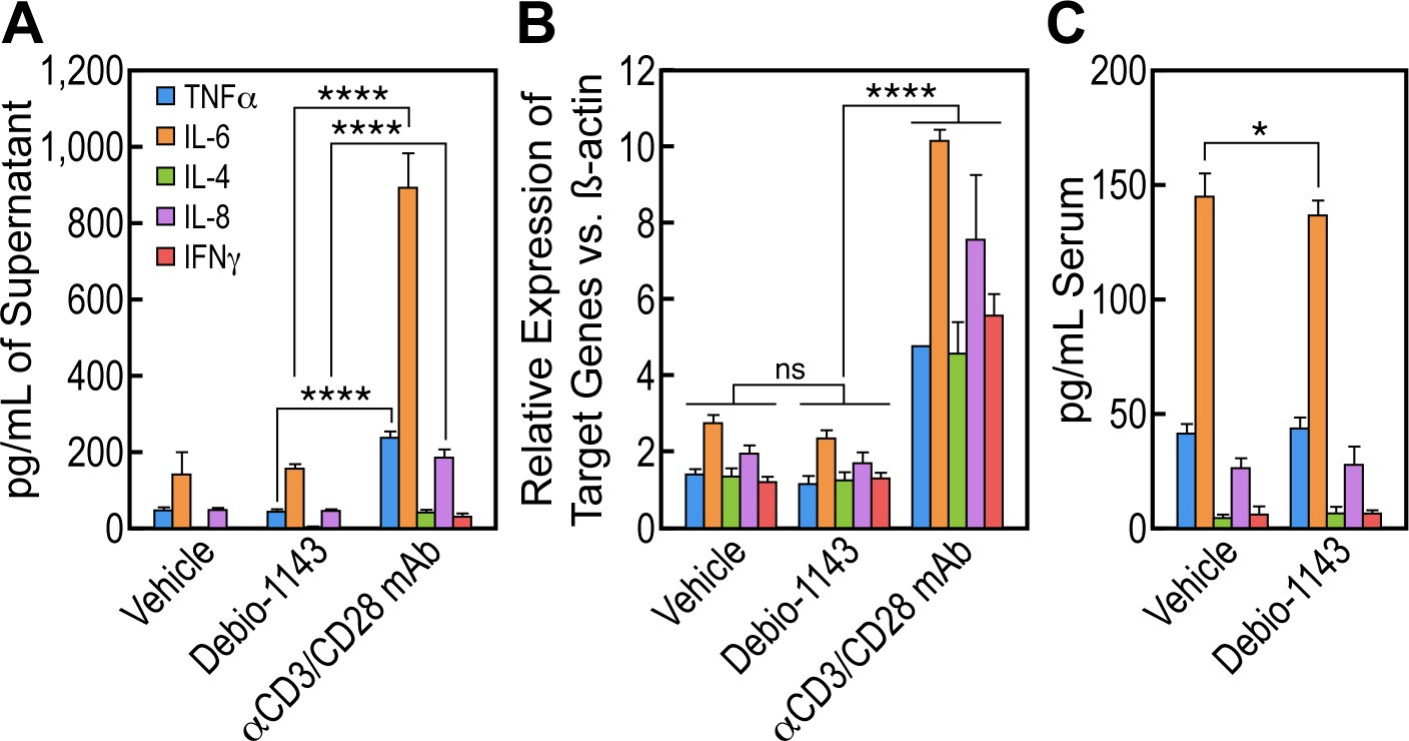
Debio 1143 does not stimulate the production of pro-inflammatory cytokines. **A.** PBMCs from an ART-treated patient were incubated with DMSO, anti-CD3 (200 ng/mL)/CD28 (500 ng/mL) antibodies (mAbs) or Debio 1143 (1 μM). Supernatants were collected after 24 h, and cytokine concentrations were determined by BioPlex analysis. **B.** Cytokine mRNAs levels from PBMCs were quantified by PCR using β-actin as control. **C.** Blood of HIV-1-infected BLT mice under ART untreated (n = 10) or treated with Debio 1143 (100 mg/kg, p.o.) (n = 10) was collected 48 h post-drug treatment and human cytokine levels were quantified by BioPlex analysis. P-values are presented. ANOVA and Bonferroni’s multiple comparison tests were used to compare means of different groups. Bar graphs indicate each sample point, mean, and SEM. Key for adjusted p-values from Bonferroni’s multiple comparison tests on graphs: ns = no significance, * = 0.01≤p<0.05, ** = 0.001 ≤p<0.01, *** = 0.0001≤p<0.001, and **** = p<0.0001.

IAPa treatment induces an increase in cytokines and chemokines that are mechanistically related to NF-kB signaling modulation in patients [63–65]. We thus examined whether Debio 1143 stimulates cytokines production in PBMCs and BLT mice. PBMCs from an ART-treated patient were treated with vehicle, anti-CD3/CD28 IgG and 1 μM Debio 1143 and analyzed for cytokines production at protein and mRNA levels. In contrast to anti-CD3/CD28 mAb, Debio 1143 failed to stimulate production of pro-inflammatory cytokines at protein (Fig 6A) or mRNA level (Fig 6B) in the tested conditions. Similarly, Debio 1143 treatment (100 mg/kg p.o. single dose) in BLT mice did not change blood levels of cytokines (Fig 6C) in the tested conditions, although one cannot exclude transient overexpression of cytokines or potential increase after multiple dosing.

## Discussion

### The IAPa Debio 1143 activates the non-canonical NF-kB signaling pathway

IAPa represent a promising new class of anti-cancer therapeutics that may foster better tumour responses to chemo/radiotherapy by creating conditions in which apoptosis can proceed. [27]. In various clinical trials, IAPa exhibited satisfactory safety and pharmacokinetic/pharmacodynamics profiles [32, 50–52]. The IAPa Debio 1143 (also called AT-406 or SM-406), which is used in the present study and which antagonizes cIAP1, cIAP2, and XIAP [50], is currently tested in various clinical trials for its anti-cancer efficacy in combination with chemo/radio-therapy, or immune checkpoint blockade in patients with advanced solid malignancies (NCT02022098, NCT03270176). These clinical trials should determine the broad spectrum of the anti-cancer efficacy and safety of the IAPa Debio 1143.

In the present study, we evaluated a property of Debio 1143, which is entirely distinct from its anti-cancer property, its capacity at reversing HIV-1 latency. We obtained evidence that Debio 1143 promotes HIV-1 transcription by activating NF-kB signaling. We first showed that Debio 1143 activates at a nM range the HIV-1 LTR by acting on NF-kB enhancer elements. We found that Debio 1143 triggers degradation of a negative regulator of the non-canonical NF-kB signaling–BIRC2 (cIAP1). This is in accordance with the previous notion that the direct binding of the IAPa to BIRC2 could trigger its auto-ubiquitination and degradation [31]. We found that Debio 1143-mediated BIRC2 degradation in CD4+ T cells is rapid (<15 min) and dose-dependent (nM range). We also found that the Debio 1143-induced BIRC2 degradation results in the accumulation of NIK, the proteolytic processing of p100 into p52, and the nuclear translocation of p52 and RELB, leading to the activation of NF-kB dimers [66]. Moreover, we showed that Debio 1143 greatly enhances the binding of RELB to the HIV-1 LTR and slightly enhances the binding of RELA to the HIV-1 LTR.

Importantly, the Chanda lab obtained similar results with another IAPa—SBI-0637142 [33]. Our data suggest a concerted action of both canonical and non-canonical transcription factors in HIV-1 activation upon SMAC mimetic treatment such as Debio 1143 and SBI-0637142 treatment. Our data also suggest that the HIV-1 latency reversal activity of SMAC mimetics (i.e., Debio 1143 and SBI-0637142) is exclusively originated via the NIK-dependent non-canonical NF-kB signaling pathway. Stimulation of this pathway triggers both RELB:p52 heterodimers, and to a minor degree, RELA:p50 heterodimers, leading to the activation of the LTR-dependent transcription that is mainly, but not solely, modulated by the non-canonical NF-kB transcription factor heterodimer. Our results reveal an unacknowledged function of the non-canonical NF-kB pathway machinery, mainly RELB:p52 heterodimers, in the regulation of HIV-1 LTR-dependent transcription. Altogether our data suggest that the IAPa Debio 1143, by activating the non-canonical NF-kB signaling pathway via BIRC2 degradation, promotes the binding of RELB:p52 complexes to the HIV-1 LTR, ultimately resulting in the activation of the LTR-dependent HIV-1 transcription.

### The IAPa Debio 1143 is a potent HIV-1 latency reversal agent

After demonstrating that Debio 1143 activates the non-canonical signaling pathway by triggering rapid degradation of the negative regulator of the non-canonical NF-kB signaling–BIRC2 –resulting in activation of HIV-1 transcription, we asked whether the IAPa possesses the ability to reverse HIV-1 latency. Remarkably, we found that Debio 1143 reverses HIV-1 latency not only in HIV-1 latent cell lines (CD4+ JLat 10.6, 2D10 and 5A8 GFP reporter cell lines) *in vitro*, but also *ex vivo* in human resting CD4+ T cells derived either from ART-treated patients or from ART-treated HIV-1-infected humanized BLT mice. In HIV-1 latent reporter cell lines, we found that Debio 1143 is as efficient, even more efficient than other selected IAPa, suggesting that Debio 1143 represents a potent LRA, at least *in vitro*. It is important to emphasize that one cannot exclude the possibility that differences in potency between LRAs may be drug batch dependent. We also found that the combination of Debio 1143 with other LRAs such as vorinostat or panobinostat exhibited an additive effect on HIV-1 latency reversal, suggesting that Debio 1143 should be combined with other compounds with distinct mechanisms of action in order to activate and eliminate viral reservoirs.

### The Debio 1143-mediated HIV-1 latency reversal is BIRC2 degradation-dependent and NIK stabilization-dependent

The reversal of HIV-1 latency by Debio 1143 is BIRC2 dependent since we found that suppressing BIRC2 expression either by Debio 1143 or siRNA treatment, reverses HIV-1 latency. Furthermore, we found that preventing BIRC2 degradation, by neutralizing the proteasome machinery (MG132 treatment), counteracts the HIV-1 latency reversal by Debio 1143. Moreover, we found that the Debio 1143-mediated BIRC2 degradation leads to the cellular accumulation of NIK as well as reversal of HIV-1 latency. Supporting the notion that NIK expression represents a precondition for the activation of the non-canonical NF-kB signaling pathway by Debio 1143, we found that the IAPa fails to reverse HIV-1 latency in a NIK knockout HIV-1 latent reporter cell line. These data further suggest that Debio 1143 reverses HIV-1 latency by acting on components of the non-canonical NF-kB signaling pathway.

### Safety of Debio 1143

In a first-in-man study, the safety, pharmacokinetics and pharmacodynamics of a daily oral administration of Debio 1143 (monotherapy) was investigated in cancer patients, and showed that the IAPa was well tolerated at doses up to 900 mg daily (QDx5 every 3 weeks), elicited BIRC2 target engagement in PBMCs and skin biopsies, and induced a moderate increase in cytokine and chemokine that are mechanistically related to NF-kB signaling modulation [50, 64]. The multiple on-going clinical trials in cancer patients should further demonstrate or not the safety of Debio 1143 in humans. We observed that a single oral administration of Debio 1143 did not induce any apparent adverse events nor an increase in cytokine levels in humanized BLT mice. One cannot exclude the possibility that a transient increase in cytokines occurs after a single Debio 1143 administration or that a persistent increase in cytokines occurs during multiple daily administrations of Debio 1143 for an extended period of time.

### Conclusions

In the present study, we provide strong evidence that the IAPa Debio 1143, by initially activating the non-canonical NF-kB signaling and subsequently reactivating HIV-1 transcription, represents an attractive viral latency reversal drug. It is anticipated that a safe and effective drug regimen for the eradication of viral reservoirs will necessitate the combination of multiple agents including HIV-1 latency reversal agents (i.e., Debio 1143). Further studies are needed to investigate the safety and efficacy of such combinations at controlling viremia and eliminating viral reservoirs to support potential future clinical research.
